# Recurrent ascending cholangitis with acute pancreatitis and pancreatic atrophy caused by a juxtapapillary duodenal diverticulum: A case report and literature review

**DOI:** 10.1097/MD.0000000000021111

**Published:** 2020-07-02

**Authors:** Nasser A.N. Alzerwi

**Affiliations:** Department of Surgery, College of Medicine, Majmaah University, Ministry of Education, Al-Majmaah, Riyadh, Kingdom of Saudi Arabia.

**Keywords:** juxtapapillary duodenal diverticulum, Lemmel syndrome, transampullary common bile duct exploration, transduodenal sphincteroplasty and septoplasty

## Abstract

**Rationale:**

Intermittent combined pancreaticobiliary obstruction may lead to multiple episodes of ascending cholangitis and pancreatitis, usually due to choledocholithiasis or periampullary mass. However, one of the rare causes is periampullary or juxtapapillary duodenal diverticulum. Although duodenal diverticula are relatively common in the general population, the overwhelming majority are asymptomatic. Duodenal diverticula can cause combined pancreaticobiliary obstruction through multiple mechanisms such as stasis-induced primary choledocholithiasis, stasis-induced intradiverticular enterolith, or longstanding diverticulitis, causing stenosing fibrosing papillitis or a combination of more than one of these mechanisms. Herein, I report a case of Lemmel syndrome due to a combination of multiple mechanisms and review the available literature on the epidemiology, pathogenesis, clinical presentation, diagnostic work-up, and management of juxtapapillary duodenal diverticulum.

**Patient concerns:**

Multiple episodes of abdominal pain, jaundice, anorexia, fever, and significant unintentional weight loss.

**Diagnoses and interventions:**

Primary choledocholithiasis, recurrent ascending cholangitis, recurrent acute pancreatitis, and pancreatic atrophy due to giant juxtapapillary duodenal diverticulum, with unsuccessful endoscopic retrograde cholangiopancreatography that was completely resolved after open transduodenal sphincteroplasty and septoplasty, transampullary and transcystic common bile duct exploration and stone extraction, and duodenal diverticular inversion.

**Outcome:**

Complete resolution of combined pancreaticobiliary obstruction without recurrence for 2 years after surgery.

**Lessons:**

Surgeons should be aware of such rare syndromes to avoid misdiagnosis and delayed or inappropriate management. Furthermore, they should understand the different available operative options for cases that are refractory to endoscopic approach.

## 1 Introduction

Intermittent combined pancreaticobiliary obstruction can lead to multiple episodes of obstructive jaundice and biliary colic with or without ascending cholangitis, as well as concomitant or interval episodes of acute pancreatitis, leading to pancreatic atrophy.^[1,2]^ The main causes are choledocholithiasis or periampullary tumors; however, rare etiologies, such as periampullary or juxtapapillary duodenal diverticulum (JPDD), have been reported.^[1,3]^ Although duodenal diverticulum (DD) is a common incidental finding in the general population, it is rarely symptomatic.^[1,3]^ Nonetheless, when DD is symptomatic, it is usually either missed or presents a diagnostic and therapeutic challenge, because even though most patients with DD can be managed without surgery, a few require technically demanding operative interventions.^[1,3,4]^ Herein, I report a case of Lemmel syndrome that required surgical intervention and also review the available literature on JPDD, with respect to epidemiology, pathogenesis, clinical presentation, diagnostic and therapeutic challenges, indications and contraindications, precautions, and the advantages and disadvantages of each diagnostic and therapeutic modality.

## 2 Case presentation

A 24-year-old Indonesian woman presented to the Accidents and Emergency Department with complaints of epigastric dull aching pain radiating to the back that was aggravated on eating and relieved on fasting. She presented with scleral jaundice, dark urine, pale stool, fever, anorexia, and weight loss of 8 kg over 6 months. She also reported multiple similar episodes over the previous 2 years, for which she had visited numerous hospitals and received no precise diagnosis or treatment. She had no other gastrointestinal (GI) symptoms and no history of previous abdominal surgery. Physical examination revealed that the patient was dehydrated, tachycardic (117 bpm), and febrile (38.7°C) with no lymphadenopathy or palpable masses; however, she had a tender epigastrium and intact hernial orifices. Bowel sounds were audible and the digital rectal examination was unremarkable.

The results of her diagnostic work-up were as follows: leukocytosis (white blood cells, 20.57 × 10 9/L) with neutrophilia of 88.38%; microcytic hypochromic anemia (hemoglobin, 9.1 g/dL); thrombocytosis (510 × 10 9/L); pre-renal azotemia (serum creatinine, 115 μmol/L; urea, 15.7 mmol/L); direct hyperbilirubinemia (total bilirubin, 114 μmol/L; direct bilirubin, 98 μmol/L; alkaline phosphatase, 1058 U/L, gamma [GT = 153 U/L]); hypoalbuminemia (serum albumin), 25 g/L; mild transaminitis (aspartate transaminase, 89 U/L; alanine aminotransferase, 41 U/L); serum amylase, 58 U/L; coagulation profile within the normal range (prothrombin time, 11.7 sec; partial thromboplastin time, 32.2 sec; international normalized ratio, 1.03 sec); random blood sugar, 6.95 mmol/L; Na, 136 mmol/L; K, 3.7 mmol/L; no lactic acidosis (pH = 7.41); and lactate, 0.5 mmol/L.

**Figure 1:**
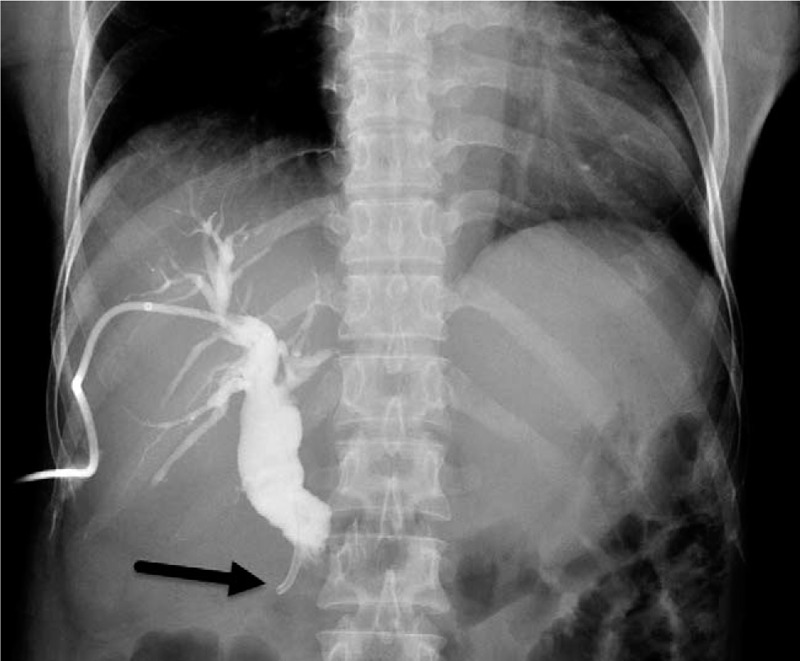
A preoperative cholangiogram through the percutaneous trans-hepatic biliary drain is shown. The procedure is performed for emergency decompression of the biliary system for the ascending cholangitis after failed endoscopic retrograde cholangiopancreatography, and the image shows failure of internal drainage (no contrast in the duodenum) due to distal obstruction at the tip and percutaneous transhepatic cholangiogram and biliary drainage in the second part of the duodenum (black arrow).

**Figure 2:**
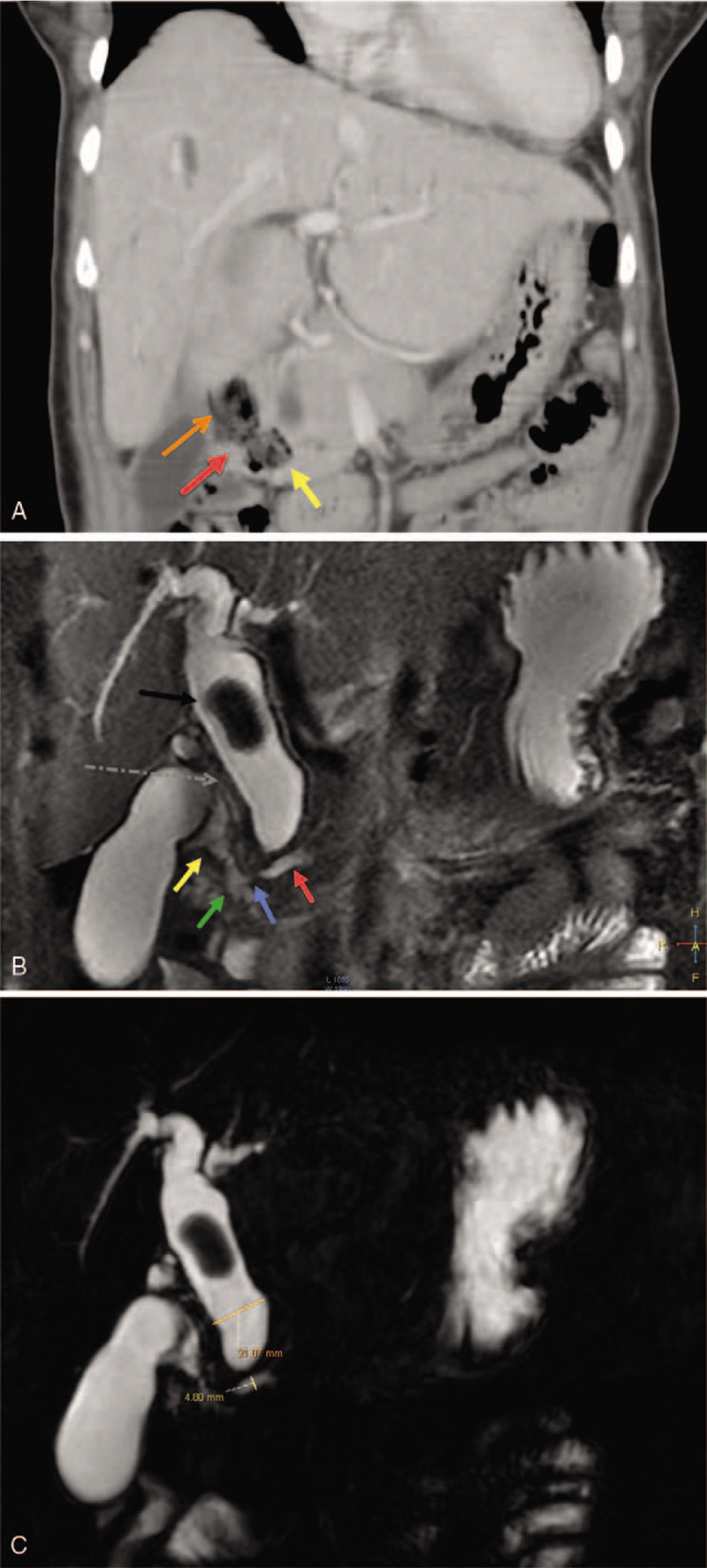
**(A)** An enhanced abdominal computed tomography scan showing coronal slice image obtained through the diverticulum (orange arrow). The narrow ostium (red arrow) connecting the diverticulum to the second part of the duodenum D2 (yellow arrow) is shown. **(B)** A magnetic resonance cholangiopancreatography image through the diverticulum showing coronal slice obtained through the diverticulum (yellow arrow). The 21 mm dilated common hepatic duct (black arrow) and an 18 mm floating choledocholith, a dilated omega-shaped cystic duct descending parallel to the common hepatic duct with low insertion to the common bile duct (dotted gray arrow), and a 4.8 mm dilated pancreatic duct of Wirsung (red arrow) with downstream stenosis (blue arrow) is shown, just before draining into the major papilla in the second part of the duodenum D2 (green arrow). **(C)** A magnetic resonance cholangiopancreatography scan of the dilated common hepatic artery showing a thick coronal slice image. A 21 mm dilated common hepatic duct (orange line), an 18 mm floating choledocholith, and a 4.8 mm dilated pancreatic duct of Wirsung (yellow line) are shown.

The abdominal X-ray was negative for pneumoperitoneum. Transabdominal ultrasonography revealed dilated extra- and intrahepatic biliary radicles (common bile duct [CBD] diameter = 23 mm; cystic duct diameter = 6 mm), with a 20 mm floating CBD stone, a dilated gallbladder (GB) with a thin wall, no pericholecystic fluid, and no stones in the GB or intrahepatic lesions. The provisional working diagnosis was ascending cholangitis due to primary choledocholithiasis (there were no GB stones and the 20 mm CBD stone could not be from a GB with a 6 mm-wide cystic duct). Following admission, she was rehydrated and treated with the administration of broad spectrum intravenous antibiotics, cefuroxime Axetil (Cefuzime Julphar, UAE) 750 mg IV in every 8 hours and metronidazole (Flagyl Sanofi-Aventis Jeddah Saudi Arabia) 500 mg IV in every 8 hours. Emergency biliary decompression was performed in the morning. Endoscopic retrograde cholangiopancreatography (ERCP) revealed a giant JPDD, and multiple attempts at identifying the papilla failed. The risk for diverticular perforation was high; hence, ERCP was aborted and the patient underwent percutaneous transhepatic cholangiography and biliary drainage (PTBD). Percutaneous transhepatic cholangiography (PTC) (Fig. 1) revealed dilated intra- and extrahepatic biliary radicles and an 18 mm CBD stone, with failure of internal drainage into the duodenum. The patient dramatically improved after biliary decompression and underwent abdominal computed tomography (CT) to further characterize the cause of the biliary obstruction (i.e., whether the primary choledocholithiasis secondary to biliary stasis was induced by biliary obstruction due to JPDD or whether the giant JPDD was just an incidental finding). CT (Fig. 2A) revealed a large stone in the common hepatic duct (CHD) with moderate intra- and extrahepatic duct dilatation, subtle hypodensity in the periampullary region, and some extraluminal air pockets. The possibility of DD or localized perforation could not be ruled out, nor could a periampullary mass.

Magnetic resonance cholangiopancreatography (MRCP) (Fig. 2B and C) was recommended to assess the relationship between the diverticulum and the papillary complex and to rule out the possibility of a periampullary mass causing the obstruction with the DD being just an incidental finding. MRCP revealed no evidence of a periampullary neoplastic mass, a periampullary diverticulum filled with air and food particles with a narrow ostium communicating with the second part of the duodenum, and a 4.8 mm dilated pancreatic duct of Wirsung with downstream stenosis at the entrance into the major papilla. MRCP also revealed dilated intra- and extrahepatic bile ducts with a single floating stone in the CHD, a distended GB with a thin wall, and an 8 mm dilated tortuous omega-shaped cystic duct with low-lying insertion into the CBD. Endoscopic ultrasonography (EUS) was not available and no biopsy was taken in the absence of a visible tumor during esophagogastroduodenoscopy (EGD)/attempted ERCP.

Even though the CBD stone was floating and she was still suffering from biliary obstruction, as well as the fact that the primary choledocholithiasis alone could not explain the recurrent acute pancreatitis leading to pancreatic atrophy, the JPDD was causing the biliary obstruction, and ERCP had failed multiple times, we offered the patient the option of surgical exploration. Based on these preoperative findings, we explained to the patient the possible operative procedures that would need to be performed, depending on the intra-operative findings. These options were double drainage (bilioenteric bypass and Roux-en-Y duodenojejunostomy or duodenal diverticulization); the Whipple procedure; or transampullary sphincteroplasty and septoplasty with combined transampullary-transcystic choledochal exploration and clearance, as well as cholecystectomy. The patient consented and was prepared for surgery.

### 2.1 Surgery

**Figure 3:**
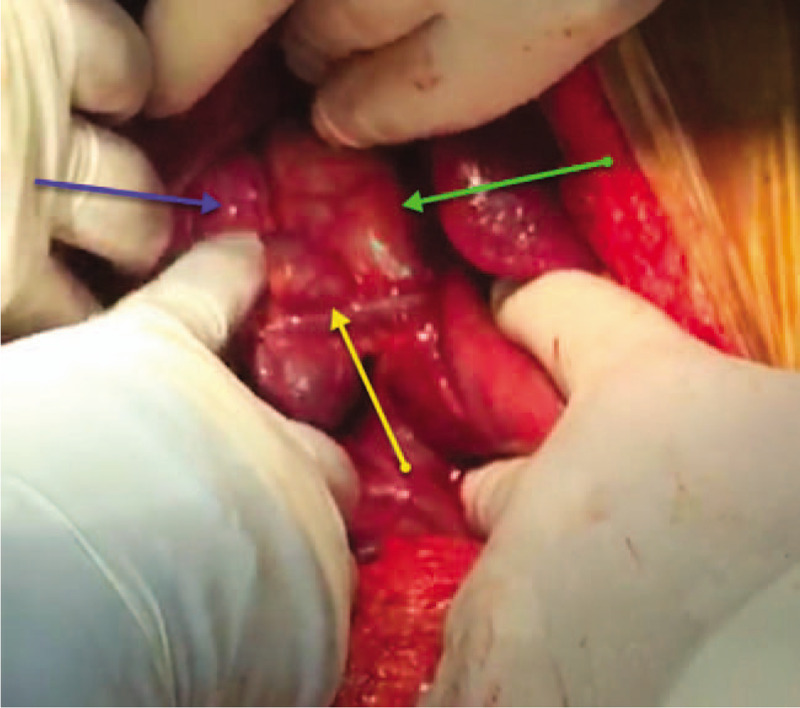
An intraoperative photo showing a 22 mm dilated common bile duct (green arrow) and an 8 mm dilated tortuous omega-shaped cystic duct (blue arrow) with low-lying insertion (yellow arrow) into the common bile duct.

**Figure 4:**
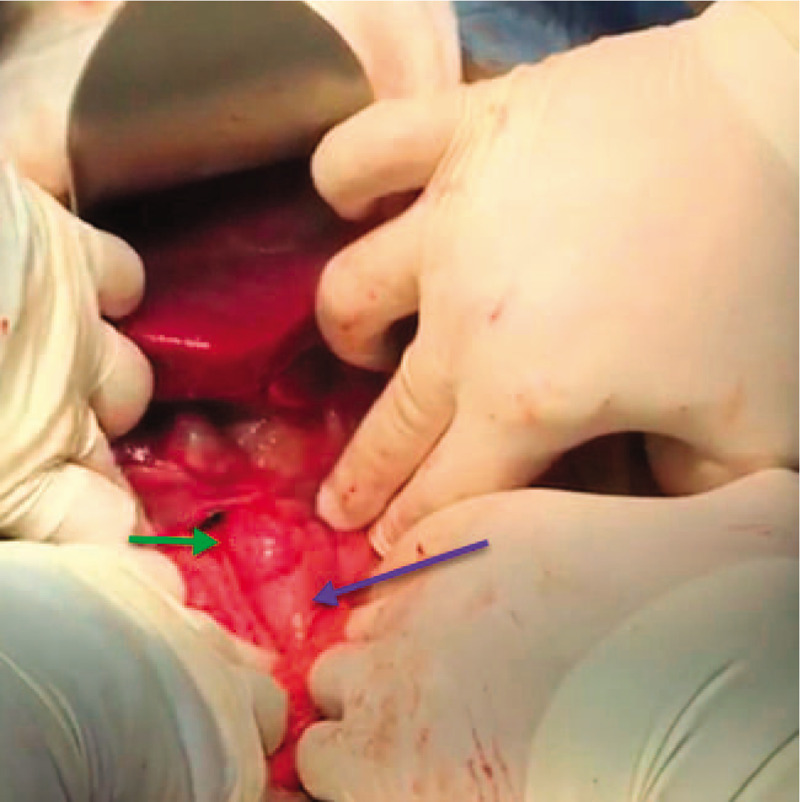
An intraoperative photo showing the second part of the duodenum (D2) (blue arrow) and the laterally protruding fundus (green arrow) of the giant (5 cm) posteromedial juxtapapillary duodenal diverticulum.

The abdominal cavity was entered through an upper midline incision with infra-umbilical extension. The findings were as follows: 1) distended GB with an 8 mm-wide tortuous, dilated, omega-shaped CD; 2) a 22 mm dilated CBD with palpable floating stone and PTBD catheter; 3) cholestatic liver (Fig. 3); and 4) a giant lateral JPDD (5 cm in diameter) (Fig. 4). Lemmel syndrome was diagnosed.

### 2.2 Actions

**Figure 5:**
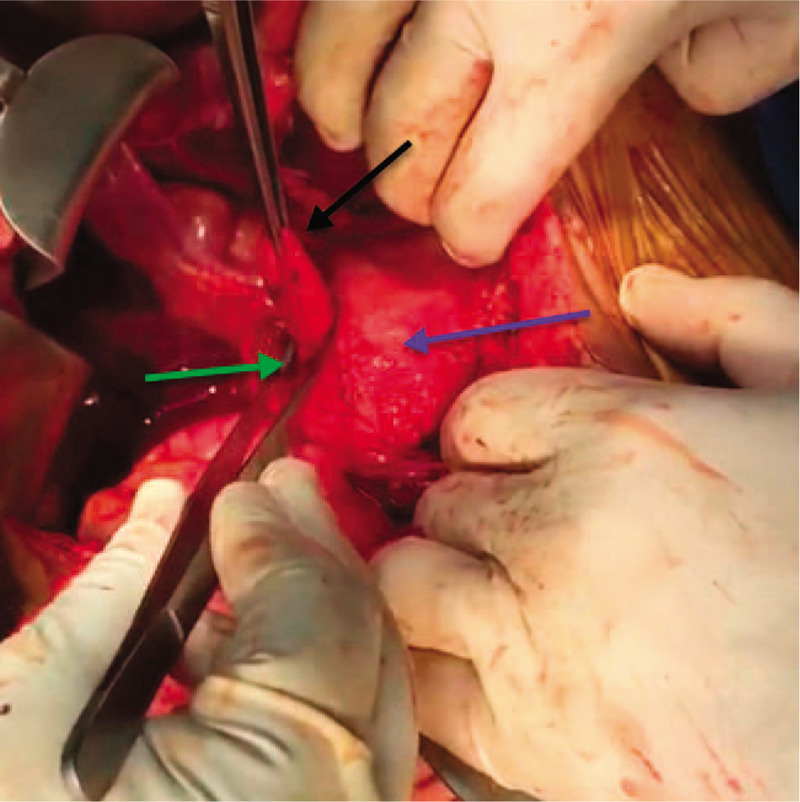
An intraoperative photo showing the kocherized second part of the duodenum (D2) (blue arrow), the neck and narrow ostium of the posteromedial juxtapapillary duodenal diverticulum (green arrow), and the fundus of the juxtapapillary duodenal diverticulum (black arrow) after opening the diverticular sac.

**Figure 6:**
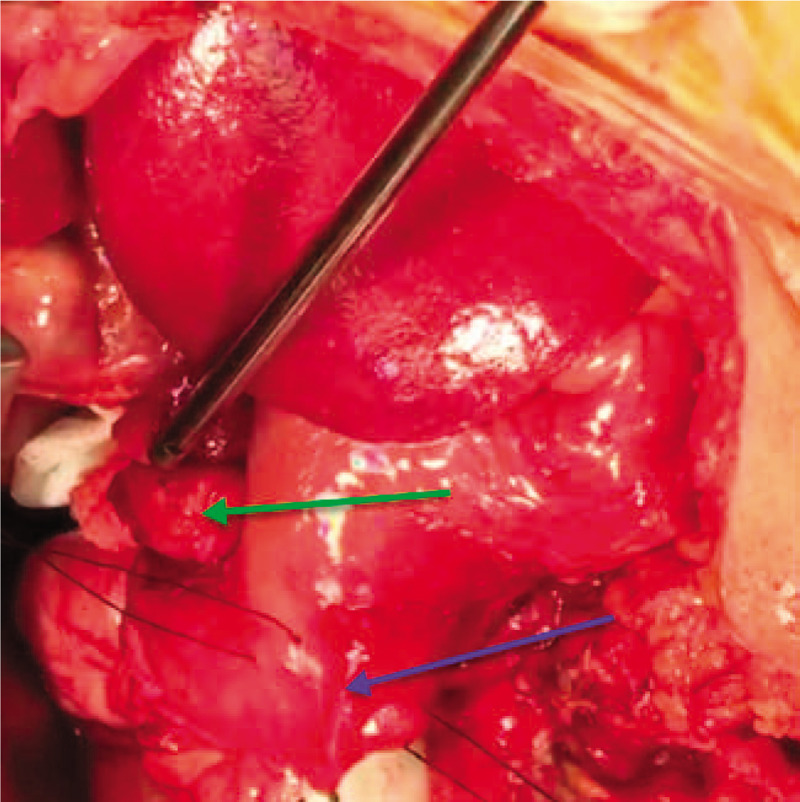
An intraoperative photo showing the kocherized second part of the duodenum (D2) (blue arrow) prepared for longitudinal duodenotomy between 2 tractional stay sutures (2.0 silk) and the fundus of the posteromedial juxtapapillary duodenal diverticulum (green arrow).

**Figure 7:**
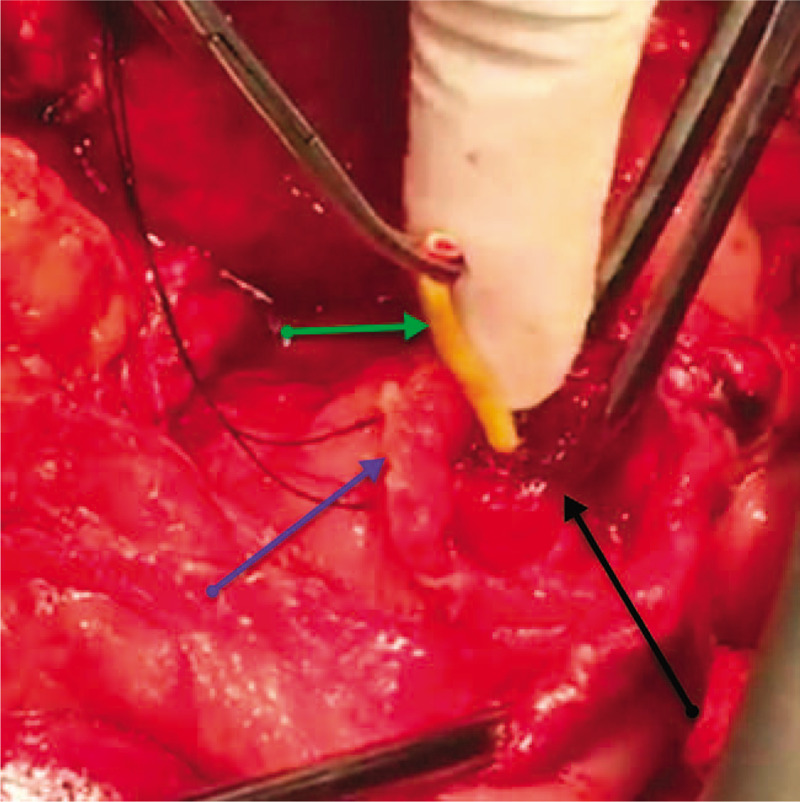
An intraoperative photo showing the longitudinally duodenotomized second part of the duodenum (D2) (blue arrow), the congested and hyperemic major papilla (black arrow), with the percutaneous transhepatic cholangiogram and biliary drainage catheter in the lumen passing through the ampulla of Vater (green arrow).

**Figure 8:**
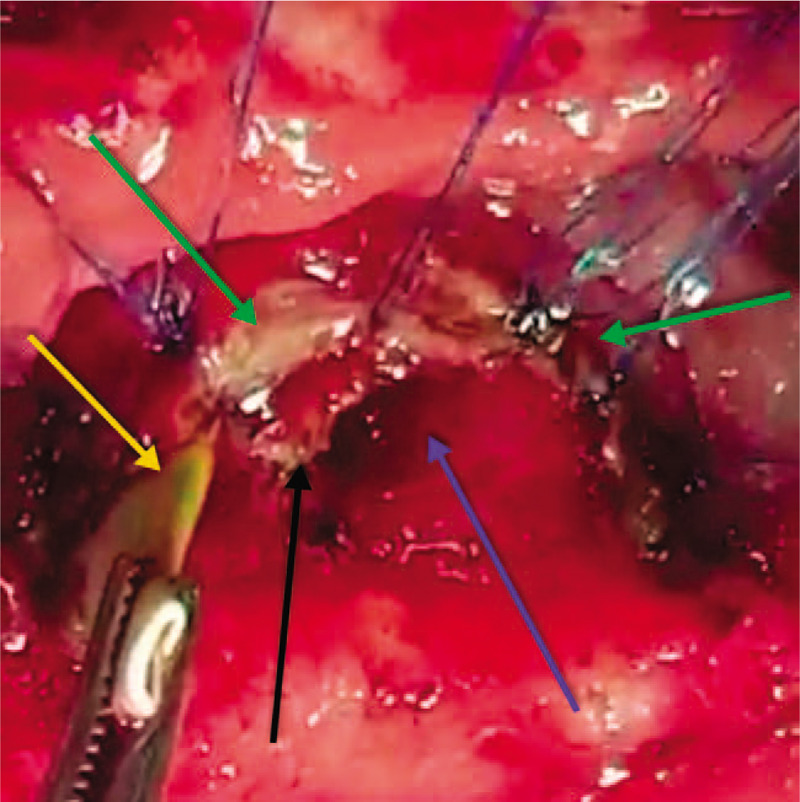
An intraoperative photo showing the completed sphincteroplasty (green arrows) using 5.0 PDS, with the percutaneous transhepatic cholangiogram and biliary drainage through the intramural common bile duct and ampulla of Vater (yellow arrow), the opening of the pancreatic duct of Wirsung (blue arrow), and the septum between the pancreatic duct and bile duct still intact (black arrow).

**Figure 9:**
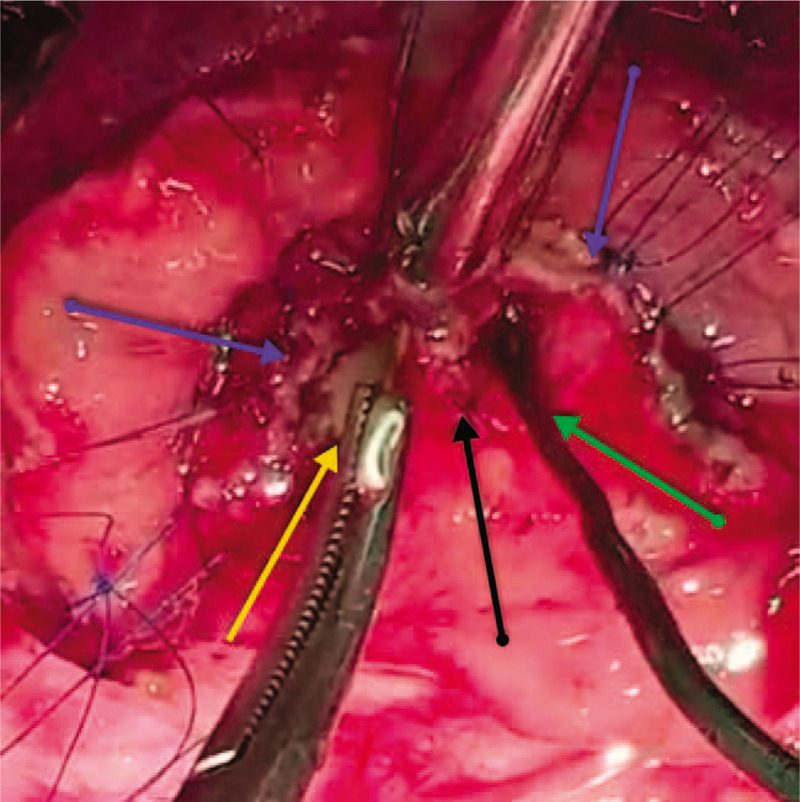
An intraoperative photo showing the completed sphincteroplasty (blue arrows) using 5.0 polydioxanone sutures, the tip of the clamp in the opening of the bile duct into the ampulla (yellow arrow), the tip of the probe in the opening of the pancreatic duct of Wirsung into the ampulla (green arrow), and the completed septoplasty of the septum between both ducts (black arrow) using 5.0 polydioxanone sutures after septectomy using monopolar cautery.

**Figure 10:**
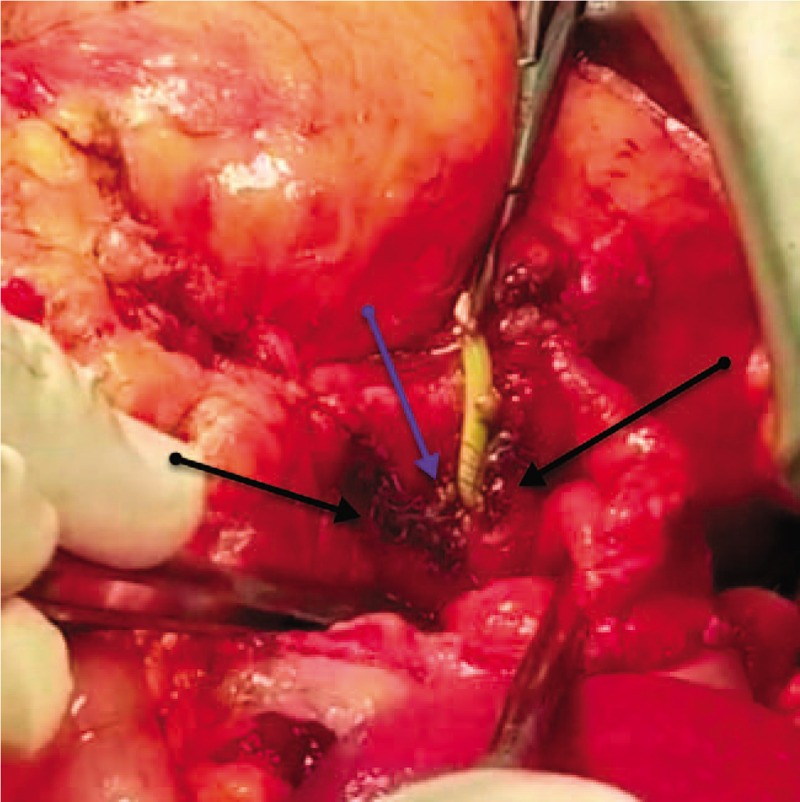
An intraoperative photo showing the completed sphincteroplasty (black arrows) and the completed septoplasty (blue arrow).

**Figure 11:**
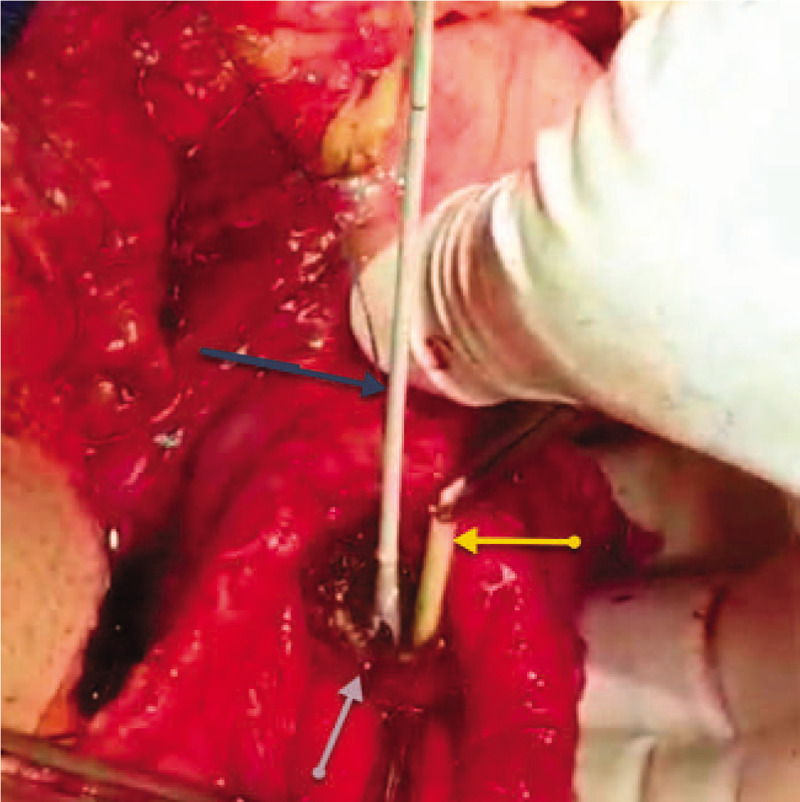
An intraoperative photo showing the completed sphincteroplasty and septoplasty (gray arrow) with the Fogarty's catheter used for trans-ampullary common bile duct exploration and clearance (dark arrow), and the percutaneous transhepatic cholangiogram and biliary drainage still in situ (yellow arrow).

**Figure 12:**
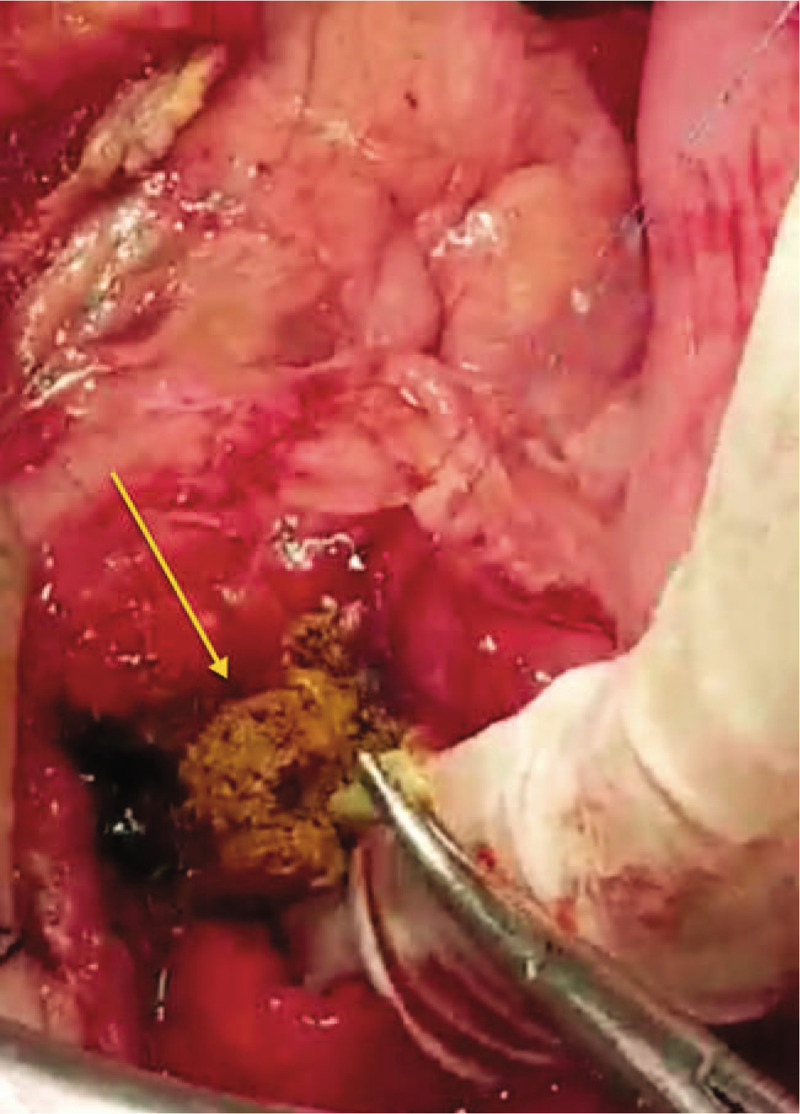
An intraoperative photo showing the trans-ampullary extraction of the crushed choledocholith (yellow arrow).

**Figure 13:**
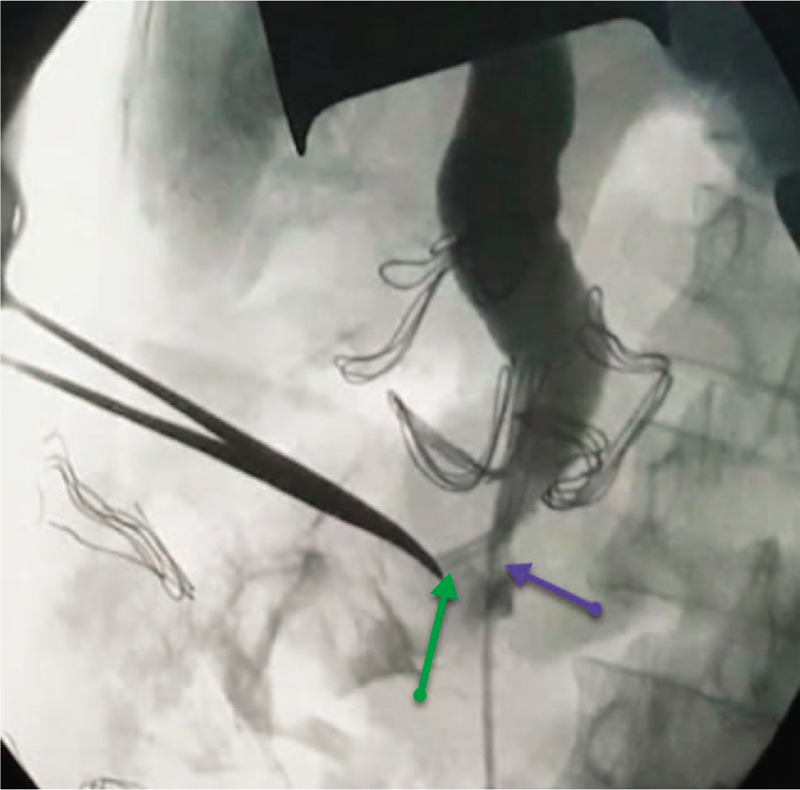
An intraoperative cholangiogram post-sphincteroplasty and trans-ampullary common bile duct exploration and clearance, showing the integrity (no extraluminal leak of contrast) and patency (no hold-up of contrast) of the sphincteroplasty. The green arrow indicates the tip of the percutaneous transhepatic cholangiogram and biliary drainage and the blue arrow indicates the Fogarty's catheter used for sweeping the common bile duct and clearing it of any debris or stones or sludge with an anterograde irrigation through the cystic duct (transcystic).

**Figure 14:**
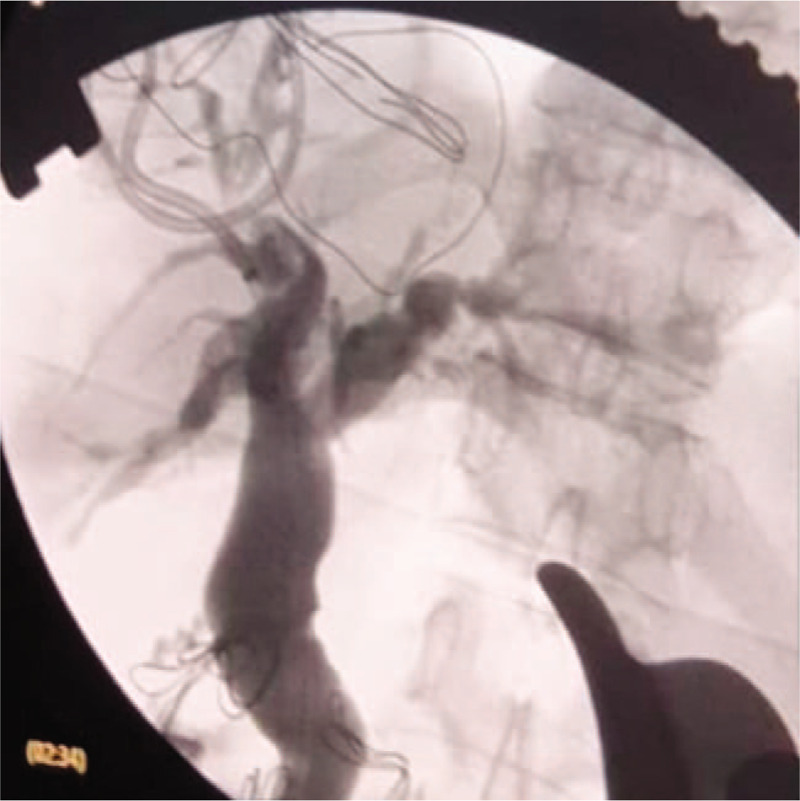
An intraoperative cholangiogram post-sphincteroplasty and trans-ampullary common bile duct exploration and clearance, showing the dilated proximal extra- and intrahepatic biliary radicles, with no filling defects or stenosis or leak.

We cautiously performed kocherization, opening the diverticular sac and dissecting the diverticulum down to its narrow-ostium neck (Fig. 5), creating a longitudinal duodenotomy of 2 cm between 2 tractional stay stitches (2.0 silk) (Fig. 6). The papilla was identified using the PTBD catheter; the papilla was inflamed, gritty, and stenotic (Fig. 7) due to long-standing diverticulitis; hence, sphincterotomy and ampullotomy were performed with extended serial sphincteroplasty and ampulloplasty stitches every 2 mm (5.0 polydioxanone [PDS], interrupted) up to 30 mm (Fig. 8). The septum between the intramural distal bile duct and the main pancreatic duct was identified (Fig. 8), and serial septotomy and septoplasty stitches (5.0 PDS, interrupted) were placed up until where the septum between the main pancreatic duct and the accessory pancreatic duct was reached (Figs. 9 and 10). Cholecystectomy was subsequently performed, followed by transcystic crushing and fragmentation of the pigment CBD stone as well as transampullary clearance of the fragments using a Fogarty's catheter and clearance of the CBD with transcystic saline irrigation (Figs. 11 and 12). Transcystic cholangiogram confirmed the patency of the CBD and the integrity of sphincteroplasty (Figs. 13 and 14). The PTBD was left in situ to prevent temporary obstruction from edema. Then, the DD was inverted, the muscle layer was closed with 2.0 Vicryl sutures, and the duodenotomy was closed transversely to avoid stenosis (2.0 Vicryl, interrupted). The dilated cystic duct was transfixed with 3.0 Vicryl and 2 prophylactic wide drains (28 French chest tubes left in situ for potential duodenal leak).

**Figure 15:**
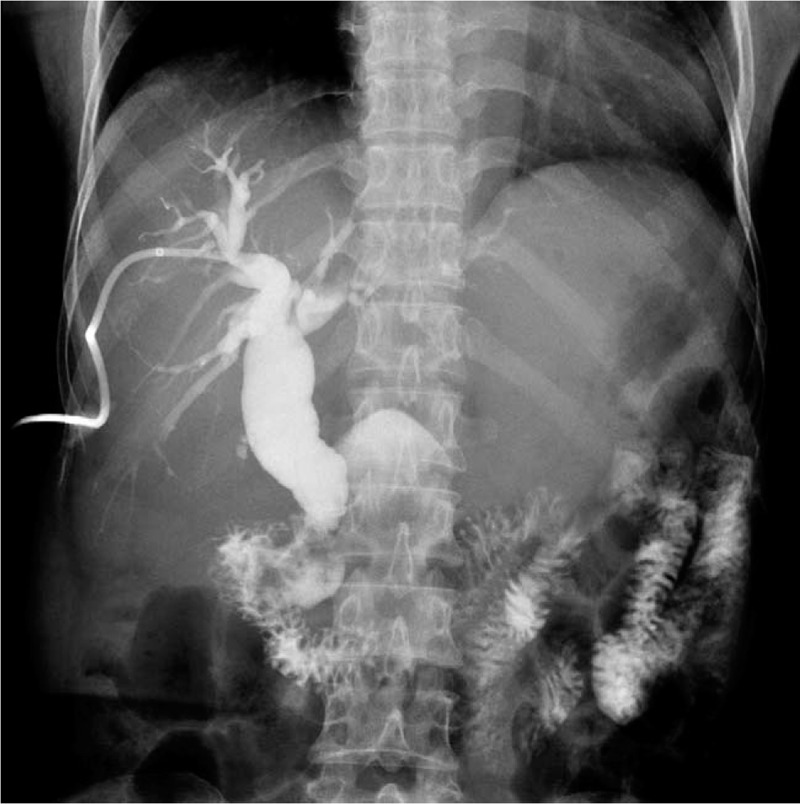
A postoperative cholangiogram on the 7^th^ postoperative day through percutaneous transhepatic cholangiogram and biliary drainage that was left in situ after surgery, showing successful internal drainage with contrast filling the small bowel immediately, and no leakage or hold-up of contrast, and no filling of the diverticulum (successful inversion of the diverticulum with no stasis in the diverticular lumen).

Postoperative recovery was uneventful, and the patient was on a free fluid diet until postoperative day (POD) 5 when she progressed to a normal diet. The drain fluid was sent for amylase and bilirubin measurement on PODs 3, 5, and 7 and was negative for biliary, pancreatic, or enteric leak. Drains were removed on POD 7 after a PTBD cholangiogram showed no leakage or hold-up of contrast with successful internal drainage (Fig. 15). The liver function test and complete blood count were normal at discharge on POD 8. After 24-month follow-up, no evidence of clinical, biochemical, or radiological recurrence was observed; furthermore, the patient confirmed the positive difference in the quality of life that she has led since the operation, with respect to complete resolution of eating-induced pain, jaundice, and anorexia, and was pleased with the weight gain.

### 2.3 Ethical approval and patient consent

The patient provided informed consent for the surgery and for publication of this report. Ethical approval was obtained (log number: 19-614E) from the King Fahad Medical City institutional review board on December 5, 2019.

## 3 Discussion and literature review

### 3.1 Epidemiology

DD was first described by Chomel in 1710 and documented by Morgagni in 1762, and the first radiological demonstration was performed by JT Case in 1913.^[5]^ Forssel and Key reported the first duodenal diverticulectomy in 1915, and Morton reported the first case series of duodenal diverticulectomy in 1940.^[6]^ With the advent of EGD and the increasing age of the population, it has become evident that DD is a common entity in the general population, with a variable prevalence ranging from 5% to 10%^[1]^ and a higher prevalence in older people, reaching 27% in octogenarians.^[3]^ Moreover, there is no sex predilection.^[6]^ Most DDs are solitary (90%) and 75% of them are periampullary (within 2–3 cm of the ampulla of Vater).^[7]^

### 3.2 Pathogenesis

The pathogenesis of DD is uncertain; however, it is most likely due to mural weakness at the vascular and ductular entry (*locus minoris resistentiae)*, supported by the clustering of DD at the perivaterian region and increasing incidence with age.^[8]^ Of patients with intra-luminal DD (IDD), 40% have intestinal malformations.^[9]^ DD can be classified as congenital or acquired, false or true, and intraluminal or extraluminal.^[8]^ The acquired false extraluminal variant is the most common. DD of the first part of the duodenum (D1) is usually tractional due to an inflammatory process.^[10]^

### 3.3 Clinical presentation

Most cases of DD are asymptomatic and discovered as an incidental finding on EGD for other purposes.^[11]^ In most patients with symptomatic DD, the DDs are periampullary^[12]^ and intraluminal.^[4]^ In our symptomatic patient, the DD was juxtapapillary and extraluminal. The clinical presentation spectrum includes the following:

- Non-specific abdominal pain or discomfort^[13]^
- Hemorrhagic presentation, which most commonly arises from the DD in the third and fourth parts of the duodenum (D3 and D4),^[10]^ most often due to ulceration from gastric acid stagnation, ectopic gastric mucosa, enterolith in wide-ostium diverticula,^[5]^ erosion into a nearby mural vessel,^[14]^ aortoenteric fistula,^[15]^ erosion of an intradiverticular polyp,^[16]^ or nonsteroidal anti-inflammatory drug intake^[17]^
- Inflammatory complications in buried narrow-ostium diverticula including diverticulitis and peridiverticulitis, spontaneous localized retroperitoneal^[18]^ or intraperitoneal perforation, free intraperitoneal perforation with generalized peritonitis,^[19]^ traumatic (accidental or iatrogenic) perforation,^[20–22]^ or duodenocolic or duodeno-jejunal fistulae^[5]^
- Enteric obstruction, which is small bowel obstruction due to enterolith^[23]^ or duodenal obstruction by the intra-luminal diverticulum^[24]^
- Isolated biliary obstruction causing recurrent episodes of extrahepatic cholestasis, primary choledocholithiasis, and/or ascending cholangitis^[25]^
- Isolated pancreatic obstruction causing recurrent episodes of acute pancreatitis and pancreatic atrophy,^[2]^ either due to stasis-induced choledocholithiasis and sludges or due to stenosing papillitis from periodic bouts of diverticulitis or intra-diverticular enterolith^[26]^
- Combined pancreaticobiliary obstruction,^[3,27,28]^ as in our patient
- Neoplastic transformation including carcinomatous and sarcomatous degeneration^[9]^

DD causing obstructive jaundice without any evidence of cholelithiasis or other secondary causes of biliary obstruction constitutes Lemmel syndrome.^[1]^ However, DD can cause primary choledocholithiasis due to stagnation and bile stasis from prolonged intermittent obstruction.^[14]^ It can also cause recurrent episodes of biliary pancreatitis and pancreatic atrophy.^[2]^ In our patient, choledocholithiasis was not the cause of biliary obstruction (it was floating and very proximal in the CHD, while the obstruction level was very distal at the ampullary level, as was the level of pancreatic obstruction that caused the dilated main pancreatic duct with pancreatic atrophy). Primary choledocholithiasis was a consequence of combined pancreaticobiliary obstruction due to juxtapapillary giant DD; hence, by definition, this was a case of Lemmel syndrome.

### 3.4 Diagnostic work-up

Diagnosis of IDD is usually made as an incidental finding on an upper GI contrast study showing the “duodenal windsock sign” (a sac filled with contrast surrounded by a thin line of radiolucency),^[29]^ whereas diagnosis of extraluminal diverticulum is usually made by contrast retention for more than 6 hours on oblique views in the erect and recumbent positions, with the presence of narrow neck differentiating it from a bulbar or post-bulbar duodenal ulcer in D1 and D2, respectively. Differentiating D4 extraluminal diverticula from gastric cancer on anteroposterior films requires a lateral film to confirm the smooth outline of the stomach.^[5]^ D3 extraluminal diverticula might be mistaken for jejunal loops.^[5]^ EGD has a sensitivity of 75% for diagnosing DD,^[30]^ and this sensitivity can be increased by using a side-viewing scope. In our patient, the diagnosis of giant JPDD was made during ERCP for emergency decompression of ascending cholangitis. EGD is notoriously known for missing D3 and D4 DD^[31,32]^ and has a sensitivity of only 30% for detecting hemorrhage from DD.^[33]^ If no other source of bleeding is evident on EGD, then angiography^[34]^ and technetium-99m-labeled red blood cell scan should be considered to increase sensitivity.^[31,33]^ The diagnosis of diverticulitis and perforation is usually made by CT and MRI showing an inter-duodenopancreatic mass with an air-fluid level and, if perforated, surrounding extraluminal free air and fluid with surrounding fat stranding.^[35–37]^ If the diverticulum is filled with fluid, it might be confused as a pancreatic cystic neoplasm.^[6,38]^ The diagnosis of DD as a cause of pancreaticobiliary obstruction, recurrent episodes of cholangitis, and pancreatitis and pancreatic atrophy is usually made using MRCP. The advantages of MRCP are that it is noninvasive and provides superior delineation of the relationship between the DD and the papilla, with superior characterization of choledochal and pancreatic ductal anatomy and pancreatic parenchymal features.^[39,40]^ In our case, MRCP confirmed the DD as the cause of combined pancreaticobiliary obstruction, ruled out neoplastic causes, and confirmed the juxtapapillary and extraluminal nature of the DD, which facilitated the decision-making regarding the operative approach. EUS is valuable in ruling out neoplastic masses as a differential of pancreaticobiliary obstruction^[41–43]^ and in facilitating papillary cannulation in intradiverticular papilla during ERCP^[44]^; however, if EUS is not available, as in our case, then MRCP should suffice. CT, PTC, and ERCP also play a crucial role in diagnosing pancreaticobiliary obstruction.^[45]^ In our case, ERCP failed to identify or cannulate the major papilla, and we resorted to PTC as an emergency biliary decompressive procedure. Transabdominal ultrasonography is limited to showing the signs of extrahepatic biliary obstruction or pancreatic parenchymal consequences of multiple episodes of acute pancreatitis (e.g., pseudocyst or significant parenchymal atrophy) as in our patient, and it is of limited value in assessment of the distal cause of such obstruction or pancreatic parenchyma.^[40]^ Plain abdominal X-ray is limited for revealing the signs of perforation, e.g., pneumoperitoneum or pneumoperitoneum, or enteric obstruction due to enterolith ileus, and it has a low sensitivity of 50%.^[19]^

### 3.5 Management

Asymptomatic and incidental DD requires no treatment or intervention.^[46]^ Non-lifestyle-limiting symptoms like nonspecific upper abdominal pain or dyspepsia also should be managed conservatively, or non-surgically with EGD for clearance of enteroliths in acute diverticular obstruction or sphincterotomy in cases of biliary obstruction.^[46]^ ERCP is the first line of management for pancreaticobiliary obstruction^[46]^ with a technical success rate over 95%.^[47]^ Although JPDD makes it technically more difficult to cannulate,^[48,49]^ it is not associated with a higher complication rate than ERCP in the absence of JPDD in terms of perforation, bleeding, or post-ERCP pancreatitis^[4]^; however, the recurrence rate is 10% to 24%.^[4]^ As elective operative management of asymptomatic or mildly symptomatic DD carries a high morbidity and a mortality risk of approximately 30%,^[5]^ and diverticulectomy has a clinical success rate of only 50% in terms of resolution of vague upper abdominal and postprandial symptoms without recurrence,^[6]^ operative intervention should be reserved for more severe cases or after failure of non-operative measures. EGD can also be helpful in the treatment of IDD by offering endoscopic diverticulectomy^[50]^ and in localizing and controlling hemorrhagic DD^[30,33]^; however, the high failure rate of localization and source control, and the high rebleeding rate of EGD,^[31–33]^ as well as the high mortality and morbidity rates following surgery makes angiography the modality of choice for localizing and controlling hemorrhagic DD.^[34]^ If EGD and angiography fail to localize or control the source of bleeding, then surgical exploration and diverticulectomy become mandatory. Only 1% to 5% of DD cases will require operative intervention for complicated DDs, such as DD perforation. Some cases can be managed non-surgically, provided the perforation is contained with no active extravasation of contrast, no generalized peritonitis, and stable hemodynamics.^[4,51]^ Non-operative management includes nil per os, nasogastric tube under suction, intravenous antimicrobials, total parenteral nutrition, close monitoring in an intensive care unit, and in cases of drainable abscesses, percutaneous radio-guided drainage with upper GI contrast studies to rule out any active extravasation of contrast before commencing oral feeding.^[4,51]^ The role of EGD in the nonsurgical management of perforated DD has been reported.^[52]^ If this non-surgical approach has failed or the patient's condition deteriorates, then operative intervention is indicated, including diverticulectomy and 2-layered closure, handsewn or stapled.^[53]^ If the duodenum is friable and there is intensive surrounding peridiverticulitis, it might be prudent to add a duodenal diverticulization procedure, and with a retroperitoneal perforation, wide drainage tubes are highly recommended.^[53]^ Preoperative diagnosis is challenging, and usually made by CT.^[36,53]^ Operative mortality is 20% to 30%,^[4,5,54]^ whereas morbidity is 30% to 40%.^[32,52]^ Nonetheless, delayed diagnosis of perforated diverticulum or delayed surgical intervention, when indicated, has a mortality rate of over 90%.^[9,55]^ Perforated JPDD should be within the surgeon's differential of perforated viscus presentation, as 14% of patients with perforated DD and generalized peritonitis have been reported to have a false negative finding on exploratory laparotomy.^[56]^ Dissection of the medial D2 JPDD is highly risky and injury to the CBD or pancreaticobiliary junction is common; potential postoperative complications include biliary, pancreatic, or duodenal fistula (13%–31%),^[9]^ especially when simple closure of the perforation is performed.^[57]^ Regarding combined pancreaticobiliary obstruction, as in our patient, once the diagnosis is confirmed, and other more serious differential diagnoses have been ruled out (e.g., periampullary carcinomas, neuroendocrine tumors), and management of the presenting emergency complications of pancreaticobiliary obstruction (e.g., ascending cholangitis or acute pancreatitis) and a non-surgical approach to decompress the pancreaticobiliary obstruction has failed to achieve long-term success with multiple recurrences, then operative management of JPDD is indicated.^[58]^ There are multiple options, ranging from bilioenteric bypass for pure biliary obstruction (e.g., side to side choledochoduodenostomy or Roux-en-Y hepaticojejunostomy); however, this option is ineffective in relieving a concomitant pancreatic obstruction, with a reported morbidity of 1% to 8% and mortality of 6%.^[4]^ For combined pancreaticobiliary obstruction, a duodenojejunostomy^[59]^ or a double drainage procedure in the form of a choledochoduodenostomy and Roux-en-Y duodenojejunostomy can be performed. This relieves biliary obstruction and a Roux-en-Y duodenojejunostomy can divert alimentary flow from the diverticulum to avoid food stagnation in the diverticulum and enterolith formation and prevent pancreatic obstruction.^[7]^ This option carries a morbidity of 21.4% and mortality of 11.6%.^[60]^ Diverticulectomy should be avoided as it carries a high risk of morbidity and mortality, with an elective mortality of 8% to 12%,^[61]^ and diverticular inversion should be considered as an alternative when necessary,^[33,62]^ as we elected to do in our patient. Laparoscopic diverticulectomy with or without intraoperative endoscopic guidance has been reported.^[63,64]^ Identification of the papilla using a transcystic guidewire is highly recommended to avoid injury to the papillary complex.^[9]^

The Whipple procedure for combined pancreaticobiliary obstruction has been reported.^[2]^ However, it carries a high morbidity of 50%^[65]^ and a mortality of 2.3%.^[66]^ Therefore, it should be reserved for cases where malignancy cannot be safely excluded or when the ampulla cannot be salvaged with the DD buried deep in the pancreatic head, or whenever there is a perforation of the diverticulum with friable D2 that does not hold stitches safely.^[67]^

One underutilized operative strategy is transduodenal sphincteroplasty and septoplasty with transampullary CBD exploration and clearance. This option relieves both biliary and pancreatic obstruction, especially when the cause of obstruction is not intra-diverticular enterolith but a stenosing fibrosing papillitis,^[68–70]^ and it allows for clearance of choledocholiths.^[71–75]^ We offered this option (along with diverticular inversion) to our patient as she was suffering from combined pancreaticobiliary obstruction due to a giant JPDD with stenosing fibrosing papillitis from longstanding stasis-induced low-grade diverticulitis with multiple failed ERCP attempts. This relieved both biliary and pancreatic obstruction and offered the potential of long-term patency compared to bypass procedures (hepaticojejunostomy, choledochoduodenostomy, duodenal diverticulization), with a lower risk of recurrence and minimal morbidity and mortality compared with resectional procedures (diverticulectomy, pancreaticoduodenectomy). However, this procedure is technically demanding and requires a lot of experience to reduce morbidity and mortality. Furthermore, experience with this procedure is decreasing, even among hepatobiliary surgeons, in the current era of EGD.^[73]^ This option has a clinical success rate of 90.5%, a recurrence rate of <0.5%, and a lower morbidity (4.9%) and mortality (1.24%) profile compared to more extensive procedures such as diverticulectomy, choledochoduodenostomy, or Whipple procedure.^[73,74]^ Careful selection is key to successful outcomes,^[72]^ as the morbidity and mortality can increase up to 30% and 5%, respectively, with poor selection or in less experienced hands.^[73]^

## 4 Conclusion

In conclusion, I have presented a case of pancreaticobiliary obstruction due to juxtapapillary DD (Lemmel syndrome) in a young Indonesian woman, which caused primary choledocholithiasis with multiple episodes of ascending cholangitis and acute pancreatitis, resulting in pancreatic atrophy. Multiple attempts at ERCP and endoscopic sphincterotomy failed, and the patient underwent successful transduodenal sphincteroplasty and septoplasty for stenosing papillitis due to long-standing and low-grade stasis-induced diverticulitis, as well as transampullary CBD exploration and clearance of choledocholiths and duodenal diverticular inversion. The patient completely recovered with no evidence of recurrence on clinical examination or on biochemical and radiological examinations after 2 years of follow-up. The management of this condition requires careful planning and decision-making as well as meticulous execution to achieve complete resolution with minimal morbidity and mortality and should be within the armamentarium of the hepatobiliary surgeon.

## Acknowledgments

The author would like to thank Dr. Suliman Al-Alshaikh, MD, Dr. Bandar Idrees, MD, Dr. Ahmed Alhumaid, MD, Dr. Khaid Alzoman, and Dr. Suliman Almutairi, MD, as well as Editage.com for their valuable input and support in proofreading and linguistic editing this manuscript. Permission to include these names in the acknowledgments was received.

## Author contributions

Idea conception, data collection, literature review, photo illustration, manuscript writing.
